# Supplementary material for: The Response of Paracoccidioides lutzii to the Interaction with Human Neutrophils
Source: J Fungi (Basel). 2023 Nov 7;9(11):1088. doi: 10.3390/jof9111088 (PMC10672145; doi:10.3390/jof9111088)
Supplement: Supplementary file 1 [file jof-09-01088-s001.zip › Figure S1.pdf]

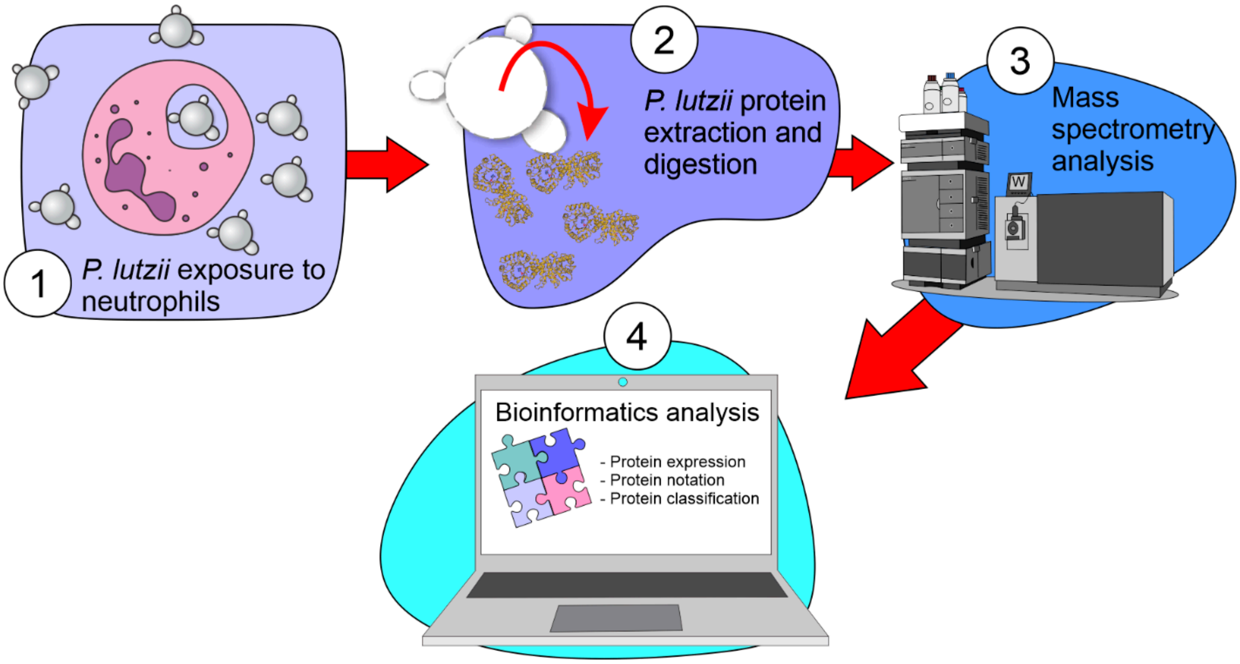

Figure S1: **Experimental workflow**. (1) *P. lutzii* yeast cells were incubated with human neutrophils for 2 hours. (2) Neutrophils were lysed and *P. lutzii* proteins were extracted and digested with trypsin. (3) Peptides were subjected to mass spectrometry analysis. (4) Bioinformatic analysis were applied to determine protein expression, notation and functional classification of the data obtained from mass spectrometry analysis.
