# Supplementary material for: The Response of Paracoccidioides lutzii to the Interaction with Human Neutrophils
Source: J Fungi (Basel). 2023 Nov 7;9(11):1088. doi: 10.3390/jof9111088 (PMC10672145; doi:10.3390/jof9111088)
Supplement: Supplementary file 1 [file jof-09-01088-s001.zip › Supplementary Table S2.pdf]

Supplementary Table S2: Increased proteins after 2 h and/or 4 h of neutrophil interaction

Accession number[a]	Description[b]	2h Score	2h Ratio[c]	4h Score	4h Ratio[c]
**Functional categories[d]**					
**METABOLISM**					
Amino acid metabolism					
PAAG_01310	2 oxoisovalerate dehydrogenase subunit alpha, mitochondrial 451 aa	1608,93	0,7558	1272,27	1,6487
PAAG_00468	4 aminobutyrate aminotransferase 286 aa	20187,1	0,7047	13068,9	1,2092
PAAG_01969	arginase 325 aa			669,95	Inf_4h
PAAG_06407	argininosuccinate lyase 300 aa			1114,38	1,3231
PAAG_03138	alanine glyoxylate aminotransferase 494 a	539,07	Inf_2h	758,76	1,2586
PAAG_08986	cystathionine beta synthase 512 aa			818,13	Inf_4h
PAAG_08701	D 3 phosphoglycerate dehydrogenase 475 aa			519,81	Inf_4h
PAAG_02361	D amino acid oxidase 335 aa	508,61	Inf_2h		
PAAG_07089	homocitrate synthase 430 aa	3129,61	0,6250	1333,6	3,5609
PAAG_03404	ketol acid reductoisomerase 134 aa			1684,66	Inf_4h
PAAG_01002	NAD specific glutamate dehydrogenase 1104 aa			1784,4	Inf_4h
PAAG_08512	serine hydroxymethyltransferase 536 aa			3558,18	1,2092
Nucleotide/nucleoside/nucleobase metabolism					
PAAG_00850	glucosamine fructose 6 phosphate aminotransferase 678 aa			971,76	Inf_4h
PAAG_05803	inosine 5 monophosphate dehydrogenase IMD2 548 aa	3894,28	0,6907	1002,1	1,4770
PAAG_08856	nicotinate nucleotide pyrophosphorylase 315 aa	4347,86	1,5527		
PAAG_01931	phosphoacetylglucosamine mutase 549 aa			508,73	Inf_4h
Nitrogen, sulfur and selenium metabolism					
PAAG_06606	cyanate hydratase 164 aa			6398,52	1,3231
PAAG_04966	hydrolase 298 aa	919,5	3,2220		

PAAG_01321	oxidoreductase 2 nitropropane dioxygenase family 368 aa			4940,96	3,2871
C-compound and carbohydrate metabolism					
PAAG_00889	phosphomannomutase 269 aa			1257,84	2,0340
PAAG_04181	sorbitol utilization protein SOU2 294 aa	677,65	1,5068	724,1	1,6161
PAAG_06817	UTP glucose 1 phosphate uridylyltransferase 507 aa	708,19	1,4333		
Lipid, fatty acid and isoprenoid metabolism					
Synthesis					
PAAG_01524	fatty acid synthase subunit beta dehydratase 2086 aa			577,72	2,6379
Oxidation					
PAAG_08859	peroxisomal multifunctional enzyme 902 aa	769,59	1,4333	523,81	Inf_4h
PAAG_02664	3 ketoacyl CoA thiolase 418 aa	13958,6	0,6250	11597,7	1,6161
PAAG_05984	glutaryl CoA dehydrogenase 429 aa	1461,68	Ctrl_2h	677,97	1,3771
Synthesis or oxidation					
PAAG_01536	trans 2 enoyl CoA reductase 415 aa			507,87	Inf_4h
PAAG_06309	enoyl CoA hydratase 298 aa	17940	1,2712	13795,2	1,3231
PAAG_08277	nitroreductase family protein 223 aa			3671,63	1,4333
Metabolism of vitamins, cofactors, and prosthetic groups					
PAAG_08166	4 hydroxyphenylpyruvate dioxygenase 404 a	14446,4	1,2337		
PAAG_00851	6 7 dimethyl 8 ribityllumazine synthase 174 aa	6124,13	1,6653		
Secondary metabolism					
PAAG_01519	inositol monophosphatase 304 aa			837,82	3,8962
**ENERGY**					
Glycolysis and gluconeogenesis					
PAAG_03243	glucose-6-phosphate 1-epimerase 318 aa			416,19	3,2871
PAAG_06172	glucokinase 517 aa			1418,68	1,2969
PAAG_02050	pyruvate decarboxylase 575 aa	9220,34	0,6570	4206,78	1,3634

PAAG_02869	phosphoglycerate kinase 418 aa			4687,95	3,9749
Tricarboxylic-acid pathway					
PAAG_03296	3 isopropylmalate dehydratase 749 aa	1133,75	Ctrl_2h	573,44	Inf_4h
PAAG_05151	ATP citrate lyase 487 aa			572,1	3,7810
PAAG_05150	ATP citrate synthase subunit 1 660 aa			717,91	3,9749
PAAG_08075	citrate synthase 475 aa			1874,99	2,0544
PAAG_00417	succinyl CoA ligase subunit alpha 332 aa	2374,55	0,6376	2530,22	1,6487
PAAG_01463	succinyl CoA ligase subunit beta 445 aa	8229,16	0,6907	1872,59	3,6693
Glyoxylate cycle					
PAAG_04542	malate synthase 540 aa	7394,14	0,7788	1344,85	2,2933
Methylcitrate cycle					
PAAG_04550	2 methylcitrate synthase 470 aa			16022,9	1,5068
Pyruvate dehydrogenase complex					
PAAG_03330	dihydrolipoyl dehydrogenase 522 aa			17342,5	1,2586
PAAG_01534	pyruvate dehydrogenase E1 component subunit beta 377 aa	14726,6	0,6376	13778,9	1,5527
PAAG_02769	pyruvate dehydrogenase protein X component 495 aa	590,7	Inf_2h	1278,94	Inf_4h
Pentose-phosphate pathway					
PAAG_01178	6 phosphogluconate dehydrogenase 492 aa			3180,28	1,3910
PAAG_04166	transaldolase 325 aa			14718,3	1,5527
Electron transport and membrane-associated energy conservation					
PAAG_07285	vacuolar ATP synthase catalytic subunit A 783 aa	2238,09	0,7334	462,71	1,9937
PAAG_06288	vacuolar ATP synthase subunit B 507 aa	979,35	Inf_2h	448,4	Inf_4h
PAAG_04838	ATP synthase subunit 4 245 aa	1455,12	Inf_2h	1379,83	Inf_4h
PAAG_06268	cytochrome c 112 aa			1373,47	1,5683
PAAG_01265	cytochrome b5 138 aa	1120,81	9,8749	4061,75	4,0148
Fermentation					

PAAG_05249	aldehyde dehydrogenase 497 aa			8839,02	1,2461
Respiration					
PAAG_00953	NADH cytochrome b5 reductase 334 aa			1041,53	Inf_4h
PAAG_03309	suaprga1 307 aa			2875,94	1,5220
**CELL CYCLE AND DNA PROCESSING**					
PAAG_08917	histone H2a 136 aa	1546,12	Inf_2h		
PAAG_07099	histone H3 3 137 aa	890,01	8,6711	1207,85	15,0293
PAAG_07098	histone H4 1 104 aa			5861,19	8,0045
PAAG_00126	histone H4 2 104 aa	2703,21	4,5267	5168,47	Inf_4h
PAAG_08918	late histone H2B L4 142 aa	2605,25	9,4877	3404,69	5,1039
PAAG_05518	cell division cycle protein 821 aa	11971,9	0,5326	6643,35	1,5068
PAAG_04458	prohibitin 1 281 aa			1116,52	3,1899
PAAG_07339	S phase kinase associated protein 1A 170 aa			3757,53	2,3869
PAAG_02522	U4/U6-U5 snRNP complex subunit LSM3 99 aa	2616,42	Inf_2h	2243,15	1,1735
**TRANSCRIPTION**					
PAAG_02255	mRNA decapping hydrolase 319 aa			1619,71	1,2214
PAAG_04571	nascent polypeptide associated complex subunit alpha 209 aa	2821,62	Inf_2h	944,78	1,3910
PAAG_00845	LSM domain containing protein 85 aa			12807,7	1,3499
PAAG_08234	transcription factor RfeF 557 aa			2637,72	1,6820
**PROTEIN SYNTHESIS**					
PAAG_08634	40S ribosomal protein S12 152 aa	1056,59	Inf_2h	1352,36	1,7160
PAAG_04690	40S ribosomal protein S15 154 aa			20017,8	1,4918
PAAG_01413	40S ribosomal protein S17 143 aa	10167	1,2712		
PAAG_03513	40S ribosomal protein S18 156 aa	12629,8	1,3634	15025,4	1,2586
PAAG_05805	40S ribosomal protein S21 89 aa	2170,98	2,2034		
PAAG_08540	40S ribosomal protein S25 158 aa	1090,62	Inf_2h		

PAAG_01785	40S ribosomal protein S3 271 aa	15397,7	1,4477		
PAAG_08955	40S ribosomal protein S3aE 256 aa			16978	1,2214
PAAG_00801	60S acidic ribosomal protein P0 lyase 314 aa			2825,87	3,0344
PAAG_07707	60S ribosomal protein L10a 230 aa			3307,02	1,7333
PAAG_05590	60S ribosomal protein L12 166 aa			7538,02	1,2969
PAAG_05379	60S ribosomal protein L17 185 aa	4533,11	1,5841	3656,81	1,6989
PAAG_08847	60S ribosomal protein L28 150 aa	845,39	Inf_2h	1341,68	1,2586
PAAG_03019	60S ribosomal protein L6 B 205 aa	4347,03	1,4049	3526,95	0,8958
PAAG_07841	60S acidic ribosomal protein P1 111 aa			33586,4	1,5527
PAAG_00240	eukaryotic translation initiation factor 5A 214 aa	3496,74	1,9348	4251,66	2,3632
PAAG_04958	eukaryotic translation initiation factor 6 248 aa			1126,62	2,1170
PAAG_06084	eukaryotic translation initiation factor 3 subunit B 737 aa	2568,27	0,6977	948,6	1,7860
PAAG_08348	eukaryotic translation initiation factor 3 39 kDa subunit 340 aa	1260,28	0,4317	728,11	Inf_4h
PAAG_00689	ATP dependent RNA helicase eIF4A 399 aa	1252,91	5,9895	2249,55	Inf_4h
PAAG_04511	ATP dependent RNA helicase SUB2 468 aa	832,16	Inf_2h		
PAAG_02024	elongation factor 1 alpha 461 aa	33947,7	1,5683	7548,89	2,2933
PAAG_03028	elongation factor 1 beta 233 aa			15183,9	1,8776
PAAG_03556	elongation factor 1 gamma 1 408 aa			2115,4	5,7546
PAAG_00594	elongation factor 2 832 aa			4047,29	3,9354
PAAG_02921	elongation factor Tu 442 aa			1874,04	1,8040
PAAG_00150	arginyl tRNA synthetase 585 aa			786,76	1,6820
PAAG_07028	histidyl tRNA synthetase 602 aa	2870,5	0,7634	915,47	2,3869
PAAG_04742	valyl tRNA synthetase 1052 aa	4052,96	0,8270	896,85	1,4770
PAAG_03951	prolyl tRNA synthetase 639 aa			494,17	Inf_4h
**PROTEIN FATE**					
PAAG_07802	proteasome component PRE6 270 aa	6777,95	1,3634	6003,98	1,2337

PAAG_03687	proteasome component PUP2 246 aa	5126,04	1,6653	3431,85	1,2712
PAAG_00664	aspartyl aminopeptidase 607 aa			920,97	1,8404
PAAG_01926	26S protease regulatory subunit 6A 462 aa			649,55	2,7732
PAAG_08931	glutamate carboxypeptidase 579 aa			6157,94	0,9704
PAAG_08184	T complex protein 1 epsilon subunit 569 aa	1590,79	Ctrl_2h	567,87	Inf_4h
PAAG_06068	T complex protein 1 subunit beta 555 aa			2039,31	Inf_4h
PAAG_01727	T complex protein 1 subunit delta 511 aa			493,75	Inf_4h
PAAG_07851	T complex protein 1 subunit eta 564 aa			595,7	Inf_4h
PAAG_07165	T complex protein 1 subunit gamma 539 aa	1708,27	2,5345	926,09	Inf_4h
PAAG_03932	E1 ubiquitin-activating protein UBA1 1030 aa			720,12	2,0751
PAAG_06536	ubiquitin 155 aa	14106,8	1,5841	30475,3	1,5068
PAAG_04901	ubiquitin conjugating enzyme 252 aa			3594,14	1,3231
PAAG_02773	ubiquitin conjugating enzyme variant MMS2 134 aa	1768,81	Inf_2h		
PAAG_03038	ubiquitin like modifier SUMO 94 aa	809,15	Inf_2h		
PAAG_09072	mitochondrial nuclease 336 aa			1052,2	2,7732
PAAG_00797	mitochondrial protein import protein MAS5 410 aa			618,62	2,4109
PAAG_07490	monothiol glutaredoxin 5 160 aa	1492,76	1,6000		
PAAG_01588	SNARE Ykt6 227 aa			507,68	Inf_4h
**CELLULAR TRANSPORT, TRANSPORT FACILITIES AND TRANSPORT ROUTES**					
PAAG_04651	GTP binding nuclear protein GSP1 Ran 216 aa			2492,33	7,8460
PAAG_08028	GTP binding protein ypt1 202 aa			4126,31	1,2840
PAAG_02458	GTP binding protein ypt7 206 aa	1749,94	5,1552	2446,88	Inf_4h
PAAG_07773	cyclin dependent kinases regulatory subunit 113 aa	839,65	Inf_2h		
PAAG_06233	vesicular fusion protein SEC17 293 aa			1208,71	1,3499
PAAG_06751	DNA damage checkpoint protein rad24 266 aa			36294,6	1,5373
PAAG_00773	DNA damage checkpoint protein rad24 293 aa			24464,7	1,7507

PAAG_08697	myosin 2 2021 aa	504,3	Inf_2h	733,47	Inf_4h
PAAG_03188	nuclear movement protein nudC 199 aa	13017,4	0,7483	12124,3	1,2461
PAAG_02186	nuclear segregation protein Bfr1 505 aa	1144,93	1,6000	583,73	1,3364
PAAG_07564	mitochondrial outer membrane protein porin 214 aa	1497,73	1,7160	1514,36	Inf_4h
PAAG_07900	phosphatidylinositol phosphatidylcholine transfer protein 353 aa			951,69	Inf_4h
PAAG_04276	phosphatidylinositol transporter 461 aa			2291,86	1,5373
PAAG_08082	plasma membrane ATPase 930 aa			536,84	1,5683
**CELLULAR COMMUNICATION/SIGNAL TRANSDUCTION MECHANISM**					
PAAG_06344	rab GDP dissociation inhibitor 469 aa			2510,33	Inf_4h
PAAG_00782	small COPII coat GTPase sar1 190 aa	2941,92	2,5345	2766,75	Inf_4h
PAAG_07634	small GTPase RhoA 192 aa	22225,1	0,7047	14067,6	1,2214
PAAG_01280	ser Thr protein phosphatase family protein 754 aa			707,54	Inf_4h
**BIOGENESIS OF CELLULAR COMPONENTS**					
PAAG_00564	actin 390 aa	604,25	Inf_2h		
PAAG_02396	actin 441 aa	3607,76	0,6065	736,48	Inf_4h
PAAG_01347	actin cytoskeleton protein VIP1 267 aa			8839,02	1,4918
PAAG_03624	Arp2 3 complex subunit Arc16 314 aa	828,45	1,5068		
PAAG_08973	tropomyosin 1 162 aa	10445,4	1,4191		
**PROTEIN WITH BINDING FUNCTION OR COFACTOR REQUIREMENT**					
PAAG_08620	ADP ATP carrier protein 310 aa			1479,12	Inf_4h
PAAG_08247	calmodulin 150 aa	22326,5	1,4049	12134,5	1,6000
PAAG_05827	glycolipid transferprotein HET C2 209 aa	623,08	1,9937	392,21	3,1899
PAAG_06917	KH domain RNA binding protein 365 aa			424,32	2,3632
PAAG_04814	nucleic acid binding protein 274 aa	2334,7	1,3771		
PAAG_00244	polyadenylate binding protein 823 aa			1008,67	2,4843
PAAG_01097	PolyrC binding protein 496 aa			691,9	Inf_4h

**CELL RESCUE, DEFENSE AND VIRULENCE**					
Stress response					
PAAG_08059	heat shock protein 60, mitochondrial 593 aa	63936,1	1,1735	94762,2	1,2586
PAAG_05679	heat shock protein 90 696 aa	10124,2	1,4049	2445,18	8,7583
PAAG_02130	heat shock protein HSP98 927 aa	454,65	Inf_2h	495,7	Inf_4h
PAAG_07775	heat shock protein SSB1 558 aa			4119,21	1,8404
PAAG_06811	heat shock protein STI1 579 aa	4905,25	1,2712	5090,56	1,5841
PAAG_01262	hsp70 like protein 629 aa			9929,8	2,0751
PAAG_05226	Hsp90 binding co chaperone Sba1 197 aa			1082,55	1,2712
PAAG_02686	Hsp90 co chaperone AHA1 330 aa	603,4	Ctrl_2h	445,02	2,0959
PAAG_08260	Hsp90 co chaperone Cdc37 508 aa	3215,48	1,6989	1060,07	2,8577
Detoxification					
PAAG_01454	peroxissomal catalase 290 aa	3725,29	1,7860	4819,99	1,1503
PAAG_02725	superoxide dismutase [Fe-Mn] 228 aa			2586,35	3,7810
PAAG_02926	superoxide dismutase [Fe-Mn] 249 aa			965,4	1,4191
PAAG_04164	superoxide dismutase [Cu-Zn] 155 aa	2192,1	1,5683	3412,51	2,3396
**UNCLASSIFIED**					
PAAG_00932	dynein light chain 95 aa			1512,92	Inf_4h
PAAG_03152	CobW domain containing protein 434 aa			436,09	3,3535
PAAG_08313	L-PSP endoribonuclease family protein Hmf1 125 aa	37221,2	1,2214		
PAAG_01399	NAD dependent epimerase/dehydratase family protein 286 aa			2069	1,8221
PAAG_07573	predicted protein 141 aa			671	Inf_4h
PAAG_03537	lysine decarboxylase-like protein 251 aa			4968,03	1,4623
PAAG_05701	putative transformer-SR ribonucleo protein 308 aa	945,03	Inf_2h		
PAAG_08058	cofilin 197 aa			2125,96	3,6693
PAAG_05019	Hnt1 cyclin dependent kinase Kin28 interacting protein 137 aa	11759,5	0,7483	18701,9	1,3910

PAAG_01591	hypothetical protein 149 aa	4091,65	1,5220		
PAAG_00772	hypothetical protein 286 aa			1008,22	Inf_4h
PAAG_03092	hypothetical protein 587 aa	2800,76	0,8106	3054,46	1,3910
PAAG_00573	hypothetical protein 71 aa	562,59	Inf_2h		
PAAG_06462	hypothetical protein 106 aa	1273,78	Inf_2h	5079,83	1,7507
PAAG_01158	conserved hypothetical protein 121 aa	3760,38	1,3910		
PAAG_03129	conserved hypothetical protein 144 aa			1470,99	Inf_4h
PAAG_07875	conserved hypothetical protein 161 aa	4399,39	1,6487	3836,2	1,2461
PAAG_05856	conserved hypothetical protein 168 aa			3799,36	1,2586
PAAG_00340	conserved hypothetical protein 174 aa			3253,81	1,3231
PAAG_00350	conserved hypothetical protein 196 aa			514	Inf_4h
PAAG_06955	conserved hypothetical protein 289 aa			2405,82	1,5527
PAAG_04274	conserved hypothetical protein 309 aa			1751,7	1,5068
PAAG_07989	conserved hypothetical protein 485 aa			680,3	1,9937

[a] Accession number from FungiDB Database (https://fungidb.org/fungidb/app, accessed on 26 September 2022).
[b] Protein description according to FungiDB Database (https://fungidb.org/fungidb/app, accessed on 26 September 2022).
[c] Expression values of differentially expressed *P. lutzii* proteins after 2 h and 4 h of neutrophil contact compared to those that were not incubated with neutrophils. Expression pattern was obtained via ProteinLynx Global Server and normalized with Phosphorylase B. Inf_2h and Inf_4h indicates that the protein was identified only after 2 h or 4 h, respectively, of neutrophil incubation compared to control without contact.
[d] Biological process according to FungiDB (https://fungidb.org/fungidb/, accessed on 26 September 2022), Uniprot (https://www.uniprot.org, accessed on 28 September 2022) and KEGG (https://www.genome.jp/pathway.html, accessed on 28 September 2022) databases.
