# Supplementary material for: The Response of Paracoccidioides lutzii to the Interaction with Human Neutrophils
Source: J Fungi (Basel). 2023 Nov 7;9(11):1088. doi: 10.3390/jof9111088 (PMC10672145; doi:10.3390/jof9111088)
Supplement: Supplementary file 1 [file jof-09-01088-s001.zip › Supplementary Table S3.pdf]

Supplementary Table S3: Decreased proteins after 2 h and/or 4 h of neutrophil interaction

Accession number[a]	Description[b]	2h		4h	
		Score	Ratio[c]	Score	Ratio[c]
**Functional categories[d]**					
**METABOLISM**					
Amino acid metabolism					
PAAG_02975	2,4-dihydroxyhept-2-ene-1,7-dioic acid aldolase 259 aa	1888,58	0,5712		
PAAG_07563	asparagine synthetase 579 aa	1206,64	Ctrl_2h	553,21	Ctrl_4h
PAAG_02603	aspartate aminotransferase 430 aa	8696,34	0,6005		
PAAG_00221	acetolactate synthase 674 aa	5089,81	0,8025		
PAAG_02163	acetyl-/propionyl-coenzyme A carboxylase alpha chain 1243 aa	933,69	Ctrl_2h	522,64	Ctrl_4h
PAAG_07114	argininosuccinate synthase 414 aa	5785,46	0,7634		
PAAG_01563	aromatic L amino acid decarboxylase 546 aa	1304,2	Ctrl_2h		
PAAG_05005	anthranilate synthase component 1 556 aa	2234,65	0,5273		
PAAG_08668	anthranilate synthase component 2 772 aa	1416,12	0,5220		
PAAG_03045	aminomethyltransferase 491 aa	8640,31	0,5655	6447,76	0,6703
PAAG_08405	cystathionine beta lyase 448 aa			415,33	Ctrl_4h
PAAG_04701	cystathionine gamma lyase 419 aa	2216,06	Ctrl_2h		
PAAG_05851	cysteine desulfurase 501 aa	1622,22	Ctrl_2h	1386,87	Ctrl_4h
PAAG_08649	cysteine dioxygenase 220 aa			951,94	Ctrl_4h
PAAG_05860	glutamate cysteine ligase 283 aa	819,8	Ctrl_2h		
PAAG_03506	glutamate decarboxylase 517 aa			472,41	Ctrl_4h
PAAG_02070	glutaminase A 904 aa	895,23	Ctrl_2h	1125,27	Ctrl_4h
PAAG_07003	glutamine synthetase 359 aa			632,28	Ctrl_4h
PAAG_04525	glutamine synthetase 487 aa	967,69	Ctrl_2h		

PAAG_05253	delta-1-pyrroline-5-carboxylate dehydrogenase 573 aa	16182,9	0,6703	11454,1	0,7945
PAAG_05776	dihydroxy acid dehydratase 606 aa			6103,76	0,6703
PAAG_08163	fumarylacetoacetase 429 aa			6443,64	0,7261
PAAG_08585	thiosulfate sulfurtransferase 345 aa	1136	Ctrl_2h		
PAAG_09095	ATP phosphoribosyltransferase 328 aa	778,5	Ctrl_2h		
PAAG_07659	chorismate synthase 374 aa	1086,82	Ctrl_2h	540,46	Ctrl_4h
PAAG_01568	glycine dehydrogenase 1184 aa	4837,58	0,7483	3537,55	0,7945
PAAG_05406	histidine biosynthesis trifunctional protein 870 aa	5149,11	0,7483		
PAAG_00106	histone acetyltransferase type B catalytic subunit 555 aa	565,48	Ctrl_2h		
PAAG_01929	HNRNP arginine N methyltransferase 308 aa	4303,5	Ctrl_2h	2303,04	Ctrl_4h
PAAG_01991	homoserine dehydrogenase 373 aa	4785,64	0,6250		
PAAG_04348	homoserine kinase 359 aa	925,73	0,6907		
PAAG_06215	hydroxymethylglutaryl CoA lyase 357 aa	997,93	0,6839	1858,6	0,7118
PAAG_04102	isovaleryl CoA dehydrogenase 431 aa	9310,11	0,6313		
PAAG_08162	maleylacetoacetate isomerase 158 aa	976,1	Ctrl_2h	5213,06	0,3296
PAAG_04103	methylcrotonoyl CoA carboxylase beta chain 582 aa			6011,44	0,6703
PAAG_08100	O acetylhomoserine thiol lyase 440 aa			28652,2	0,6250
PAAG_06431	ornithine aminotransferase 461 aa	10648,1	0,7711		
PAAG_07317	PENR2 protein 314 aa	784,59	Ctrl_2h		
PAAG_03237	phospho 2 dehydro 3 deoxyheptonate aldolase 487 aa			490,52	Ctrl_4h
PAAG_06096	phospho 2 dehydro 3 deoxyheptonate aldolase 373 aa	595,13	Ctrl_2h		
PAAG_07243	phosphoribosylformylglycinamidine synthase 1362 aa	837,86	Ctrl_2h	721,79	Ctrl_4h
PAAG_01302	phosphorylase family protein 312 aa			2321,98	0,8781
PAAG_02693	saccharopine dehydrogenase 451 aa	2258,2	0,6250		
PAAG_02354	serine 3 dehydrogenase 249 aa	27845,5	0,4630		
PAAG_07412	serine hydroxymethyltransferase 472 aa	7760,84	0,6703		

Nucleotide/nucleoside/nucleobase metabolism					
PAAG_07312	CTP synthase 587 aa	790,76	Ctrl_2h		
PAAG_04974	adenylosuccinate lyase 485 aa			1806,65	0,7634
PAAG_02170	adenylosuccinate synthetase 422 aa			3111,03	0,7408
PAAG_07428	allantoinase 553 aa	727,67	Ctrl_2h		
PAAG_08152	3 5 bisphosphate nucleotidase 353 aa			398,06	Ctrl_4h
PAAG_05611	deoxyuridine 5 triphosphate nucleotidohydrolase 217 aa	2163,92	0,5273		
PAAG_05392	betaine aldehyde dehydrogenase 479 aa			5498,65	0,6977
PAAG_00731	bifunctional purine biosynthesis protein ADE17 626 aa	18815	0,5655		
PAAG_07529	orotidine 5 phosphate decarboxylase 162 aa			1489,66	0,7788
PAAG_03530	phosphoribosylamine glycine ligase 805 aa	619,65	Ctrl_2h	862,62	Ctrl_4h
Nitrogen, sulfur and selenium metabolism					
PAAG_00954	urease 837 aa			687,22	Ctrl_4h
PAAG_06237	urease accessory protein ureG 271 aa	1079,66	Ctrl_2h		
PAAG_07811	sulfite oxidase 449 aa	3031,88	0,7483		
Phosphate metabolism					
PAAG_00661	4 nitrophenylphosphatase 308 aa	1009,37	Ctrl_2h	371,08	Ctrl_4h
C-compound and carbohydrate metabolism					
PAAG_03978	3 hydroxyisobutyrate dehydrogenase 345 aa	6701,68	0,6250		
PAAG_05780	2 5 diketo D gluconic acid reductase A 280 aa	1291,25	0,6440		
PAAG_04233	2 nitropropane dioxygenase 331 aa	602,78	Ctrl_2h		
PAAG_04526	3 beta hydroxysteroid dehydrogenase 276 aa			955,22	Ctrl_4h
PAAG_05761	4 carboxymuconolactone decarboxylase family protein 186 aa	765,12	Ctrl_2h		
PAAG_00685	alpha mannosidase 1098 aa	3444,93	0,7558		

PAAG_07804	D lactate dehydrogenase 525 aa	1058,05	0,7483		
PAAG_01885	galactokinase 529 aa	849,84	Ctrl_2h		
PAAG_05690	esterase D 287 aa			1740,25	0,8106
PAAG_07276	glycogen synthase 711 aa	866,96	Ctrl_2h	552,26	Ctrl_4h
PAAG_06473	mannitol 1 phosphate 5 dehydrogenase 389 aa	6950,11	0,7558	10457,5	0,7711
PAAG_04602	mannosyl oligosaccharide glucosidase 850 aa	733,45	0,6376		
PAAG_08351	isocitrate dehydrogenase [NADP], mitochondrial 492 aa			1413,59	Ctrl_4h
PAAG_04290	neutral alpha glucosidase AB 970 aa			603,25	Ctrl_4h
PAAG_02115	ribose phosphate pyrophosphokinase 455 aa	1080,21	Ctrl_2h		
Lipid, fatty acid and isoprenoid metabolism					
Synthesis					
PAAG_01525	fatty acid synthase subunit alpha reductase 1883 aa	773,66	Ctrl_2h		
PAAG_04963	oleate induced peroxisomal protein 130 aa	4624,27	Ctrl_2h		
PAAG_02150	acyl CoA thioesterase II 356 aa	581,45	Ctrl_2h	403,03	Ctrl_4h
PAAG_01870	3-oxoacyl-[acyl-carrier protein] reductase 295 aa	1087,92	Ctrl_2h	539,53	Ctrl_4h
Oxidation					
PAAG_03116	acyl coenzyme A oxidase 683 aa	699,66	Ctrl_2h		
PAAG_06224	carnitine O acetyltransferase 611 aa			2634,4	0,6771
PAAG_06175	peroxisomal matrix protein 167 aa			23537,1	0,8270
PAAG_07746	3 ketoacyl CoA thiolase 422 aa	1286,87	Ctrl_2h		
PAAG_03689	3 ketoacyl CoA thiolase B 420 aa	11777,1	0,5655	9649,39	0,6188
PAAG_05454	acyl CoA dehydrogenase 440 aa	11957,5	0,7118	12158,1	0,6839
PAAG_07786	acetyl CoA acetyltransferase 400 aa	9270,93	0,7711		
PAAG_03447	acetyl CoA acetyltransferase 439 aa	4561,71	0,7047		
PAAG_00435	R benzylsuccinyl CoA dehydrogenase 445 aa			1050,16	Ctrl_4h
Synthesis or oxidation					

PAAG_06329	3 hydroxybutyryl CoA dehydrogenase 322 aa	10656,3	0,7788		
PAAG_01833	2 succinylbenzoate CoA ligase 583 aa	1419,62	Ctrl_2h		
PAAG_07631	short chain dehydrogenase reductase family protein 256 aa	1347,16	Ctrl_2h		
PAAG_05093	succinyl CoA 3 ketoacid coenzyme A transferase subunit B 515 aa	10251	0,6005	7115,22	0,8106
PAAG_03216	mitochondrial peroxiredoxin PRX1 223 aa	7533,36	0,7711	11639,6	0,5379
Metabolism of vitamins, cofactors, and prosthetic groups					
PAAG_04294	methylenetetrahydrofolate reductase 714 aa	1726,81	Ctrl_2h	730,66	Ctrl_4h
PAAG_07036	methylmalonate semialdehyde dehydrogenase 586 aa	14768,5	0,5379		
PAAG_00293	quinone oxidoreductase 203 aa			857	Ctrl_4h
PAAG_02382	quinone oxidoreductase 392 aa	5822,92	0,5945	4744,35	0,7788
PAAG_01934	riboflavin synthase alpha chain 239 aa			3779,56	Ctrl_4h
PAAG_08349	C 1 tetrahydrofolate synthase 940 aa	906,97	Ctrl_2h		
Secondary metabolism					
PAAG_04888	4 coumarate CoA ligase 583 aa	1354,96	Ctrl_2h		
PAAG_01324	folic acid synthesis protein 601 aa			3430,48	0,6313
PAAG_07591	diphosphomevalonate decarboxylase 406 aa	1029,09	Ctrl_2h		
PAAG_03960	isopentenyl diphosphate Delta isomerase 272 aa	1187,53	Ctrl_2h	1642,61	Ctrl_4h
**ENERGY**					
Glycolysis and gluconeogenesis					
PAAG_02011	phosphoglucomutase 763 aa	2299,35	0,7945	2308,03	0,6839
PAAG_01015	hexokinase 428 aa			1743,78	0,8106
PAAG_03643	6 phosphofructo 2 kinase 535 aa	745,82	Ctrl_2h		
PAAG_08468	glyceraldehyde 3 phosphate dehydrogenase 338 aa	52210,4	1,7333	56727,1	0,7788
PAAG_01995	fructose bisphosphate aldolase 361 aa	19801,4	0,7788		
PAAG_02585	triosephosphate isomerase 250 aa	17069,2	0,8187	22836,5	0,8025

PAAG_08203	phosphoenolpyruvate carboxykinase 578 aa	5972,56	0,7408	9191,5	0,6440
PAAG_06380	pyruvate kinase 535 aa	30539,4	0,5169	22075,3	0,8187
Tricarboxylic-acid pathway					
PAAG_07843	aconitate hydratase 801 aa	585,32	Ctrl_2h		
PAAG_04559	2 methylcitrate dehydratase 562 aa			59883,6	0,8025
PAAG_05328	3 isopropylmalate dehydrogenase A 363 aa	606,42	Ctrl_2h	555,77	Ctrl_4h
PAAG_05048	3 isopropylmalate dehydratase large subunit 786 aa	17412,9	0,6126	16139,1	0,6376
PAAG_00726	pyruvate carboxylase 1197 aa	2817,76	0,7945		
PAAG_02732	2 oxoglutarate dehydrogenase E1 1073 aa	2180,27	0,6907		
PAAG_01725	succinate dehydrogenase flavoprotein subunit 672 aa	2090,06	0,7711	6260,18	0,6313
PAAG_06103	succinate dehydrogenase iron sulfur subunit 283 aa			1936,28	Ctrl_4h
PAAG_00588	fumarate hydratase 364 aa	4810,51	0,7047		
PAAG_07729	isocitrate dehydrogenase subunit 2 342 aa	1188,02	Ctrl_2h		
Glyoxylate cycle					
PAAG_06951	isocitrate lyase 538 aa			6624,44	0,8106
PAAG_04549	mitochondrial 2 methylisocitrate lyase 602 aa	8962,62	0,7483		
Pentose-phosphate pathway					
PAAG_05940	deoxyribose phosphate aldolase 280 aa			666,92	Ctrl_4h
PAAG_04444	transketolase 686 aa	8021,92	0,8353		
PAAG_01481	FGGY family carbohydrate kinase 617 aa	674,31	Ctrl_2h		
Electron transport and membrane-associated energy conservation					
PAAG_06155	vacuolar ATP synthase subunit E 240 aa			2523,86	Ctrl_4h
PAAG_03668	vacuolar ATP synthase subunit F 125 aa			1009,06	Ctrl_4h
PAAG_05605	ATP synthase delta chain 166 aa	4776,94	Inf_2h	9150,56	0,6637
PAAG_05576	ATP synthase gamma chain 298 aa	16473,7	0,6313	17824,8	0,6637
PAAG_06206	ATP synthase subunit 5 231 aa	788,81	Ctrl_2h		

PAAG_08037	ATP synthase subunit beta 514 aa			59337,6	0,6505
PAAG_06093	cytochrome c1 323 aa	946,96	Ctrl_2h		
PAAG_07672	ubiquinol-cytochrome c reductase subunit 7 122 aa	7656,62	Ctrl_2h	7804,24	Ctrl_4h
PAAG_06796	cytochrome c oxidase subunit 4, mitochondrial 197 aa	620,05	Ctrl_2h		
Fermentation					
PAAG_00403	alcohol dehydrogenase 355 aa	4878,41	0,7945		
PAAG_03910	aldehyde dehydrogenase 472 aa	1304,33	Ctrl_2h		
Respiration					
PAAG_00173	electron transfer flavoprotein subunit alpha 349 aa	6869,75	0,5886		
PAAG_04931	electron transfer flavoprotein subunit beta 263 aa	5092	0,7788	8865,75	0,7118
**CELL CYCLE AND DNA PROCESSING**					
PAAG_08471	histone H2A Z 139 aa	2068,3	Ctrl_2h		
PAAG_02230	cell division control protein 193 aa			1476,71	Ctrl_4h
PAAG_08074	clock controlled gene 9 protein 711 aa	961,82	Ctrl_2h		
PAAG_06511	mitochondrial genome maintenance protein mgm101 295 aa	2533,01	Ctrl_2h		
PAAG_00923	proliferating cell nuclear antigen 282 aa			22991,9	0,8270
**TRANSCRIPTION**					
PAAG_03652	cap binding protein 832 aa	1193,21	Ctrl_2h		
PAAG_03941	G4 quadruplex nucleic acid binding protein 442 aa	842,83	Ctrl_2h		
PAAG_03783	GAF domain nucleotide binding protein 213 aa	1660,08	Ctrl_2h		
PAAG_04496	nascent polypeptide associated complex subunit beta 168 aa			618,01	Ctrl_4h
PAAG_01710	polymerase II polypeptide D 153 aa	1128,23	Ctrl_2h		
PAAG_03710	mRNA splicing protein SME1 94 aa	1190,8	Ctrl_2h		
PAAG_06565	pre mRNA splicing factor ini1 111 aa			1629,41	Ctrl_4h
PAAG_04637	pre mRNA splicing factor rse1 1210 aa	668,62	Ctrl_2h		

PAAG_01736	ran GTPase activating protein 498 aa	798,38	Ctrl_2h		
PAAG_02466	ran specific GTPase activating protein 478 aa	22261,5	0,5220	30250,1	0,7788
PAAG_01630	small nuclear ribonucleoprotein LSM2 99 aa	3522,85	0,6505		
PAAG_02537	small nuclear ribonucleoprotein Sm D2 115 aa	2601,98	Ctrl_2h	986,06	0,2019
PAAG_03030	splicing factor U2AF associated protein 399 aa	692,93	Ctrl_2h		
PAAG_02437	U2 small nuclear ribonucleoprotein B 254 aa	1660,54	Ctrl_2h		
PAAG_00847	RNA methylation protein TRM1125 aa			553,61	Ctrl_4h
PAAG_01042	cleavage stimulation factor subunit 2 306 aa			521,1	Ctrl_4h
**PROTEIN SYNTHESIS**					
PAAG_06367	30S ribosomal protein S17P 162 aa	5693,42	0,4771		
PAAG_02111	40S ribosomal protein S0 296 aa	16049,9	0,7047	13499,5	0,7334
PAAG_05017	40S ribosomal protein S10 A 166 aa	3359,26	0,8353		
PAAG_05704	40S ribosomal protein S13 1 152 aa	11821,5	0,7334	8064,04	0,7408
PAAG_01433	40S ribosomal protein S14 152 aa	6079,82	0,6839		
PAAG_01435	40S ribosomal protein S16 144 aa			5681,03	0,7408
PAAG_09043	40S ribosomal protein S2 261 aa	8377,41	0,4771	9507	0,6771
PAAG_05337	40S ribosomal protein S22 131 aa	7360,93	0,7945	15699,5	0,4916
PAAG_00385	40S ribosomal protein S23 146 aa			803,7	Ctrl_4h
PAAG_06882	40S ribosomal protein S24 135 aa	8118,43	Ctrl_2h	13835	Ctrl_4h
PAAG_07847	40S ribosomal protein S26 122 aa	16543,8	0,6005	18525,8	0,7711
PAAG_03816	40S ribosomal protein S4 263 aa	6643,12	0,5886		
PAAG_07182	40S ribosomal protein S7 202 aa			32138,4	0,6005
PAAG_00264	40S ribosomal protein S8 B 203 aa	12069,5	0,5117		
PAAG_03828	40S ribosomal protein S9 192 aa	4421,29	0,8187		
PAAG_02553	54S ribosomal protein YmL6 347 aa	2764,95	Ctrl_2h	777,5	Ctrl_4h
PAAG_01052	60S ribosomal protein L10 B 224 aa			25149,9	0,7408

PAAG_06320	60S ribosomal protein L13 218 aa	4642,12	0,2276		
PAAG_00969	60S ribosomal protein L15 204 aa	4015,15	Ctrl_2h	7902,53	0,5769
PAAG_07955	60S ribosomal protein L18 190 aa	6431,56	0,4274	10181,9	0,4630
PAAG_08497	60S ribosomal protein L19 192 aa	10343,8	Ctrl_2h		
PAAG_00430	60S ribosomal protein L2 255 aa	14064,5	0,2808	19318,6	0,5599
PAAG_00952	60S ribosomal protein L20 175 aa	9764,44	0,5016		
PAAG_03827	60S ribosomal protein L21 A 161 aa	18895,7	0,4232	19542,2	0,7634
PAAG_07385	60S ribosomal protein L23a 154 aa	9688,31	0,2698	10275,4	0,4966
PAAG_05233	60S ribosomal protein L26 135 aa	2696,66	Ctrl_2h	5792,09	Ctrl_4h
PAAG_01939	60S ribosomal protein L27 A 136 aa	2327,65	Ctrl_2h	6572,29	Ctrl_4h
PAAG_00088	60S ribosomal protein L3 393 aa	4382,07	0,7047		
PAAG_06792	60S ribosomal protein L34 B 121 aa	3747,94	Ctrl_2h	3029,95	0,4538
PAAG_08888	60S ribosomal protein L4 A 373 aa			15630,9	0,3135
PAAG_06569	60S ribosomal protein L43 93 aa			9897,64	Ctrl_4h
PAAG_06487	60S ribosomal protein L7 C 249 aa	7181,62	0,6907	7797,62	0,6637
PAAG_04998	60S ribosomal protein L8 B 262 aa			13287,6	0,5599
PAAG_00347	60S ribosomal protein L9 B 193 aa	8087,53	0,7334	11490,7	0,8106
PAAG_00205	60S ribosomal protein L24 145 aa			7876,76	Ctrl_4h
PAAG_01001	40S ribosomal protein S29 81 aa	1071,41	Ctrl_2h		
PAAG_09096	40S ribosomal protein S28 69 aa	25278	0,2393	27799,5	0,4538
PAAG_00724	ribosomal protein 11 large subunit 176 aa			11840	0,7118
PAAG_06743	ribosomal protein L23e 141 aa	6577,25	Ctrl_2h	4262,78	Ctrl_4h
PAAG_02889	ribosomal protein L35 126 aa	1027,6	Inf_2h	1900,46	Ctrl_4h
PAAG_00765	60S ribosomal protein L36 176 aa	16777,2	0,4190	11635,9	0,7408
PAAG_06970	mitochondrial 37S ribosomal protein RSM24 376 aa	828,99	Ctrl_2h		
PAAG_01731	mitochondrial 54S ribosomal protein YmL37 219 aa	785,65	Ctrl_2h		

PAAG_01050	cytosolic large ribosomal subunit protein L30 109 aa	7281,94	0,6005	13153,2	0,6250
PAAG_03167	elongation factor G 1 798 aa	586,09	Ctrl_2h		
PAAG_01777	alanyl tRNA synthetase 963 aa	6313,91	0,6065		
PAAG_07349	glycyl tRNA synthetase 656 aa	1396,12	0,7261		
PAAG_03572	glutaminyl tRNA synthetase 718 aa			2166,75	0,7408
PAAG_07105	isoleucyl tRNA synthetase 1080 aa	1101,87	Ctrl_2h		
PAAG_04193	cysteinyl tRNA synthetase 821 aa	752,73	Ctrl_2h		
PAAG_02865	translation initiation factor RLI1 624 aa	916,53	Ctrl_2h		
PAAG_01786	phenylalanyl tRNA synthetase beta chain 600 aa	1778,33	Ctrl_2h		
PAAG_08702	seryl tRNA synthetase 489 aa	1412,01	0,7118	4297,79	0,2491
PAAG_01711	tRNA methyltransferase Trm5 480 aa	1907,35	Ctrl_2h	891,79	Ctrl_4h
**PROTEIN FATE**					
PAAG_00768	peptidase family protein 443 aa			807	Ctrl_4h
PAAG_05788	peptidyl prolyl cis trans isomerase A2 251 aa	7680,58	0,5945	7493,82	0,6637
PAAG_00739	peptidyl prolyl cis trans isomerase B 211 aa			9887,13	0,4148
PAAG_06168	peptidyl prolyl cis trans isomerase cypE 142 aa			5713,62	0,7634
PAAG_03334	peptidyl prolyl cis trans isomerase D 374 aa			34318,9	0,8270
PAAG_01778	peptidyl prolyl cis trans isomerase H 181 aa	1819,66	Ctrl_2h		
PAAG_09004	puromycin sensitive aminopeptidase 899 aa	3918,75	0,6126	3272,5	0,7866
PAAG_07500	xaa Pro aminopeptidase 469 aa	1043,43	Ctrl_2h		
PAAG_07319	xaa Pro aminopeptidase 699 aa	2462,73	Ctrl_2h	1265,21	Ctrl_4h
PAAG_07467	dipeptidyl peptidase 773 aa	2361,19	0,8187	1231,73	0,8270
PAAG_00986	disulfide isomerase Pdi1 539 aa	27315,5	0,7483		
PAAG_05643	NAD(P)H:quinone oxidoreductase, type IV 220 aa	20623,6	0,5886		
PAAG_05107	AAA ATPase 921 aa	579,59	Ctrl_2h		
PAAG_00238	ubiquitin 219 aa	7897,75	Ctrl_2h		

PAAG_04327	ubiquitin carboxyl terminal hydrolase 1129 aa	682,86	Ctrl_2h		
PAAG_00295	ubiquitin carboxyl terminal hydrolase 784 aa	585,61	Ctrl_2h	798,37	Ctrl_4h
PAAG_00367	ubiquitin-binding protein CUE5 434 aa	1645,58	Ctrl_2h	538,82	Ctrl_4h
PAAG_07890	vacuolar sorting protein snf7 223 aa	3560,46	Ctrl_2h		
PAAG_07037	calnexin 568 aa	2454,18	0,7483		
PAAG_03533	Hsp70 nucleotide exchange factor fes1 226 aa			1203,08	Ctrl_4h
PAAG_07849	metacaspase 1 384 aa			367,55	Ctrl_4h
PAAG_01650	monothiol glutaredoxin 5 277 aa	539,84	Ctrl_2h		
**CELLULAR TRANSPORT, TRANSPORT FACILITIES AND TRANSPORT ROUTES**					
PAAG_08954	GTP cyclohydrolase 501 aa	1574,33	Ctrl_2h		
PAAG_02820	GTP dependent nucleic acid binding protein engD 394 aa	1057,48	Ctrl_2h		
PAAG_07111	cyclin dependent kinase B1-1 407 aa	647,39	Ctrl_2h		
PAAG_03953	WD repeat containing protein 425 aa	639,51	Ctrl_2h		
PAAG_01947	ATPase GET3 342 aa	684,47	Ctrl_2h		
PAAG_02019	F-type H+-transporting ATPase subunit H 219 aa			4490,94	0,7261
PAAG_06878	V-type ATPase, G subunit 140 aa			984,64	Ctrl_4h
PAAG_02467	ran-specific GTPase-activating protein 1 478 aa	2894,47	Ctrl_2h		
PAAG_03445	kinesin heavy chain 1023 aa			400,38	Ctrl_4h
PAAG_07185	actin like protein 2/3 complex, subunit 5 193 aa			612,17	Ctrl_4h
PAAG_03717	myosin regulatory light chain cdc4 140 aa	3122,03	Ctrl_2h		
PAAG_05960	NIPSNAP family protein 410 aa	9239,23	0,8106	6733,49	0,6907
PAAG_00326	iron/copper transporter Atx1 81 aa	18073,8	Ctrl_2h		
**CELLULAR COMMUNICATION/SIGNAL TRANSDUCTION MECHANISM**					
PAAG_02377	rho GDP dissociation inhibitor 186 aa	6597,71	0,7189		
PAAG_00783	serine threonine protein phosphatase PP1 325 aa	695,92	Ctrl_2h		
**BIOGENESIS OF CELLULAR COMPONENTS**					

PAAG_00004	actin binding protein 582 aa	3722,17	Ctrl_2h	1370,04	Ctrl_4h
PAAG_00997	actin interacting protein 614 aa			839,36	Ctrl_4h
PAAG_06192	ARP2 3 complex 20 kDa subunit 170 aa	3219,42	0,4317		
PAAG_05663	ARP2 3 complex 34 kDa subunit 319 aa	1268,4	Ctrl_2h		
PAAG_00622	mitochondria fission 1 protein 154 aa	661,33	Ctrl_2h		
PAAG_07150	UDP galactopyranose mutase 521 aa	2925,09	0,6839		
PAAG_06885	UDP N acetylglucosamine pyrophosphorylase 748 aa	2630,13	0,7047		
**PROTEIN WITH BINDING FUNCTION OR COFACTOR REQUIREMENT**					
PAAG_00501	ADP ribose pyrophosphatase 216 aa			1826,97	Ctrl_4h
PAAG_01452	ADP ribosylation factor 171 aa	628,13	Ctrl_2h		
PAAG_07702	ADP ribosylation factor 184 aa			4294,32	0,5712
PAAG_06574	curved DNA binding protein 42 kDa protein 419 aa	1377,89	Ctrl_2h		
PAAG_06886	zinc finger protein GIS2 223 aa			2325,13	Ctrl_4h
PAAG_03551	calcineurin subunit B 190 aa	2654,7	Ctrl_2h		
PAAG_07887	cAMP dependent protein kinase regulatory subunit 445 aa	1204,54	Ctrl_2h		
PAAG_03640	CCR4 Not complex subunit Caf16 273 aa	1252,29	Ctrl_2h		
PAAG_09122	FK506 binding protein 141 aa			4799,3	Ctrl_4h
PAAG_01424	iron binding protein erpA 113 aa	715,5	Ctrl_2h		
PAAG_02352	molybdopterin binding domain containing protein 332 aa	857,65	Ctrl_2h	625,53	Ctrl_4h
PAAG_08703	rheb small monomeric GTPase RhbA 264 aa	1260,16	Ctrl_2h		
**CELL RESCUE, DEFENSE AND VIRULENCE**					
Stress response					
PAAG_07750	heat shock protein Hsp88 728 aa	25035,6	0,7189		
PAAG_05142	10 kDa heat shock protein mitochondrial 103 aa	38028,7	0,7711	80806,1	0,5769
PAAG_08003	hsp70 like protein 655 aa			99072,4	0,7788

PAAG_03292	cytochrome c peroxidase 375 aa			14778,7	0,7047
PAAG_06947	gamma glutamyltranspeptidase 594 aa			1230,24	Ctrl_4h
Detoxification					
PAAG_07020	thioredoxin reductase 359 aa			1323,23	Ctrl_4h
**MISCELLANEOUS**					
PAAG_02802	ankyrin repeat containing protein 215 aa	780,58	Ctrl_2h		
PAAG_01704	SAM dependent methyltransferase UbiE/COQ5 family protein 301 aa			662,7	Ctrl_4h
PAAG_08992	type 2A phosphatase activator tip41 243 aa	877,44	Ctrl_2h		
**UNCLASSIFIED**					
PAAG_01935	formyl coenzyme A transferase 450 aa	1666,83	0,6703		
PAAG_06083	dienelactone hydrolase family protein 246 aa	5374,49	0,6703		
PAAG_02665	DlpA domain-containing protein 239 aa	700,98	Inf_2h	551,67	Ctrl_4h
PAAG_06515	DUF833 domain containing protein 321 aa	8513,61	Ctrl_2h	2348,46	Ctrl_4h
PAAG_02567	EF hand superfamily Ca2 modulated protein 190 aa	1014,82	Ctrl_2h		
PAAG_08179	RING superfamily domain-containing protein 231 aa	1087,27	Ctrl_2h	1015,05	Ctrl_4h
PAAG_01539	SNF7 family protein Fti1/Did2, variant 240 aa	1727,3	Ctrl_2h	484,51	Ctrl_4h
PAAG_07792	secondary thiamine-phosphate synthase enzyme 219 aa	8547,98	Ctrl_2h		
PAAG_01045	NIF3-like protein 110 aa			467,04	Ctrl_4h
PAAG_03741	hydrolase-HD superfamily protein 152 aa			4268,04	Ctrl_4h
PAAG_05864	COPII-coated vesicle protein 401 aa	690,66	Ctrl_2h		
PAAG_08693	Bot1p superfamily domain-containing protein, partial 412 aa	1882,75	Ctrl_2h		
PAAG_01572	zuotin 445 aa			2687,7	0,7634
PAAG_02434	hypothetical protein 132 aa			1321,19	Ctrl_4h
PAAG_06923	hypothetical protein 164 aa	546,88	Ctrl_2h		

PAAG_05550	hypothetical protein 173 aa			1393,39	Ctrl_4h
PAAG_03239	hypothetical protein 177 aa			2155,76	Ctrl_4h
PAAG_05348	hypothetical protein 231 aa	1636,04	Ctrl_2h	2312,75	Ctrl_4h
PAAG_01776	hypothetical protein 241 aa			674,4	Ctrl_4h
PAAG_05822	hypothetical protein 256 aa			438,42	Ctrl_4h
PAAG_05224	hypothetical protein 282 aa	2388,38	0,6440		
PAAG_01863	hypothetical protein 69 aa	2106,85	Ctrl_2h	2905,31	Ctrl_4h
PAAG_02828	hypothetical protein 79 aa	12074,8	0,4148		
PAAG_04647	conserved hypothetical protein 115 aa	573,85	Ctrl_2h		
PAAG_03925	conserved hypothetical protein 121 aa	1346,8	Ctrl_2h		
PAAG_07543	conserved hypothetical protein 121 aa			1477,94	Ctrl_4h
PAAG_07411	conserved hypothetical protein 196 aa	816,97	Ctrl_2h		
PAAG_07772	conserved hypothetical protein 211 aa			7540,36	0,6126
PAAG_01327	conserved hypothetical protein 239 aa			619,6	Ctrl_4h
PAAG_00579	conserved hypothetical protein 298 aa	754,48	Ctrl_2h		
PAAG_00904	conserved hypothetical protein 347 aa			400,54	Ctrl_4h
PAAG_08413	conserved hypothetical protein 348 aa	677,99	Ctrl_2h		
PAAG_00335	conserved hypothetical protein 370 aa	727,96	Ctrl_2h	1105,78	Ctrl_4h
PAAG_01717	conserved hypothetical protein 414 aa	3596,53	Ctrl_2h		
PAAG_02068	conserved hypothetical protein 450 aa			1746,28	Ctrl_4h
PAAG_05547	conserved hypothetical protein 686 aa			400,59	Ctrl_4h
PAAG_02004	conserved hypothetical protein 908 aa	666,05	Ctrl_2h	596,27	0,6440

[a] Accession number from FungiDB Database (https://fungidb.org/fungidb/app, accessed on 26 September 2022).

[b] Protein description according to FungiDB Database (https://fungidb.org/fungidb/app, accessed on 26 September 2022).

[c] Expression values of differentially expressed *P. lutzii* proteins after 2 h and 4 h of neutrophil contact compared to those that were not incubated with neutrophils. Expression pattern was obtained via ProteinLynx Global Server and

normalized with Phosphorylase B. Ctrl_2h and Ctrl_4h indicates that the protein was identified only in control without neutrophil contact.

[d] Biological process according to FungiDB (https://fungidb.org/fungidb/, accessed on 26 September 2022), Uniprot (https://www.uniprot.org, accessed on 28 September 2022) and KEGG (https://www.genome.jp/pathway.html, accessed on 28 September 2022) databases.
